# Spontaneous Common Femoral Artery Pseudoaneurysm: A Case Report

**DOI:** 10.31729/jnma.6948

**Published:** 2022-03-31

**Authors:** Prabhat Silwal, Robin Man Karmacharya, Satish Vaidya, Shashank Shrestha, Mahesh Mani Adhikari

**Affiliations:** 1 Cardio-thoracic and Vascular Unit, Department of Surgery, Dhulikhel Hospital, Dhulikhel, Kavre, Nepal; 2 General and Gastrointestinal Unit, Department of Surgery, Dhulikhel Hospital, Dhulikhel, Kavre, Nepal

**Keywords:** *case report*, *common femoral artery*, *misdiagnosis*, *pseudoaneurysm*, *vascular surgery*

## Abstract

Spontaneous femoral artery pseudoaneurysm in a young person with no comorbidity is a rare occurrence. A 30 years old gentleman presented to our hospital with complaints of painful swelling of spontaneous onset in the right inguinal region for 15 days. He had undergone incision and drainage of the contents of the swelling five days back but he suffered from a recurrence of the painful right inguinal swelling and persistent bleeding from the incision site for four days. Computed tomography showed a pseudoaneurysm of the right common femoral artery. It was treated surgically by emergency exploration, hematoma evacuation, removal of pseudoaneurysm, and repair of the defect in the right common femoral artery. In this case, we were fortunate that inadvertent incision of the pseudoaneurysm didn't result in a massive haemorrhage. This serves as a reminder that the possibility of a femoral artery pseudoaneurysm should be considered when evaluating a swelling of the inguinal region.

## INTRODUCTION

A pseudoaneurysm is a cavity that communicates with the lumen of an artery through a disruption on its wall.^1^ Femoral artery is reported to be the most common site.^2,3^ Common causes of femoral artery pseudoaneurysm are previous endovascular procedures and anastomotic breakdown following arterial reconstructive surgery.^1,3^ Pseudoaneurysms can resemble cutaneous abscesses owing to the presence of features of inflammation.^1^ Inadvertent incision of such swelling can result in a devastating complication. Here, we describe a rare case of spontaneous right common femoral artery pseudoaneurysm in a young person complicated by incision and drainage performed after misdiagnosis as a cutaneous abscess.

## CASE REPORT

A 30 years old gentleman presented to our hospital with complaints of painful swelling of spontaneous onset in the right inguinal region for 15 days. Five days back, he had visited a community health worker in his village. Aspiration was done from the swelling which showed dark reddish content which was removed through a 0.5 cm long incision on the skin over the swelling. Five days after undergoing the procedure, the gentleman presented to our Emergency Department with complaints of recurrence of painful swelling in the right inguinal region and bleeding from the incision site for four days. He didn't have any history of endovascular surgery, external trauma, or substance abuse. He smokes cigarettes with a pack-year of 15. He was not hypertensive and non-diabetic.

His vital signs were within the normal range. On local examination, the swelling was present in the right inguinal region, 10x5 cm in size, globular, tender to touch, and firm in consistency with smooth margins. A puncture wound of 0.5x0.2 cm was present over the swelling with reddish discharge oozing out from it. Pulsation, peristalsis, cough impulse, fluctuation, and bruits were not appreciated.

**Figure 1:**
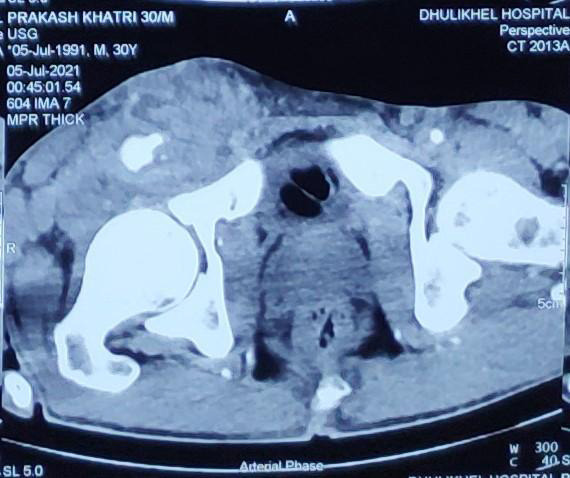
Contrast-enhanced computed tomography arterial phase showing the pseudoaneurysm (left arrow) and the right common femoral artery (right arrow).

Duplex ultrasonography and Contrast Enhanced Computed Tomography (CECT) scan showed a pseudoaneurysm of 1.7x1.5 cm in the right common femoral artery, directed superolateral with neck measuring 7x5 cm and surrounding hematoma measuring 9.2x8.8x5.2 cm (Figure 1). Right inguinal lymphadenopathy was also noted.

**Figure 2:**
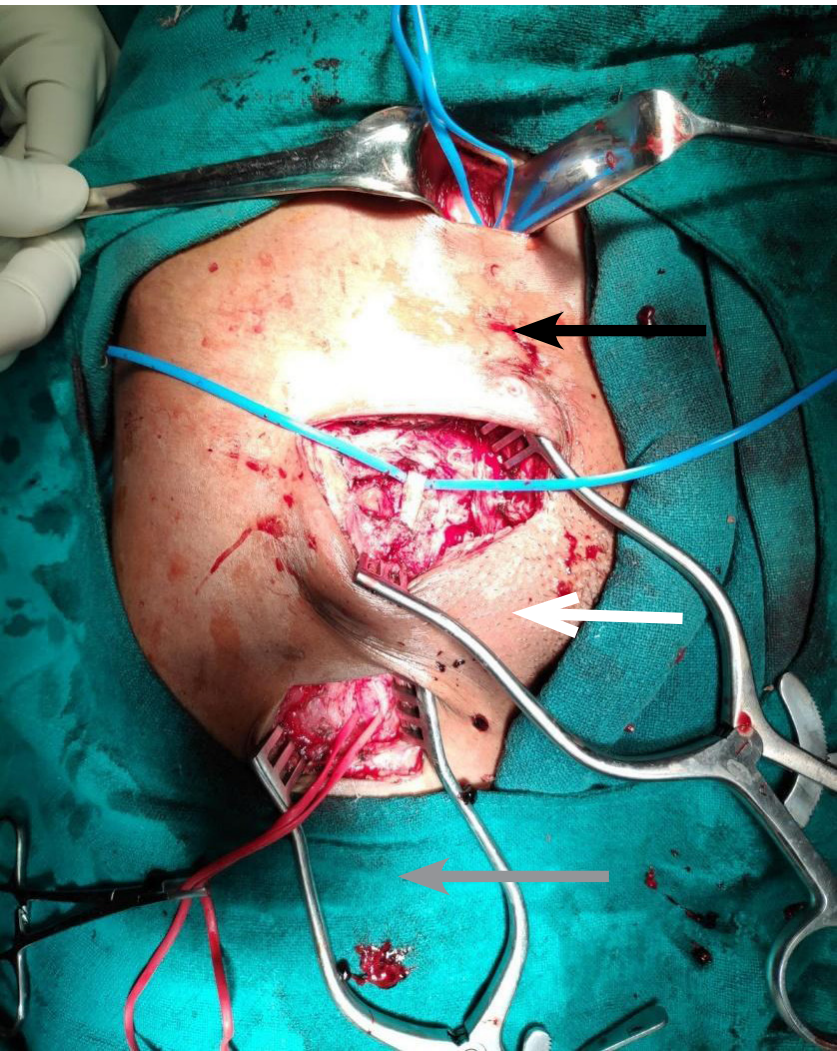
Intraoperative photograph following excision of pseudoaneurysm showing incisions for proximal control (black arrow), excision of pseudoaneurysm (white arrow) and distal control (grey arrow).

Emergency surgical intervention was planned under general anaesthesia. Three skin incisions were given (Figure 2).

**Figure 3:**
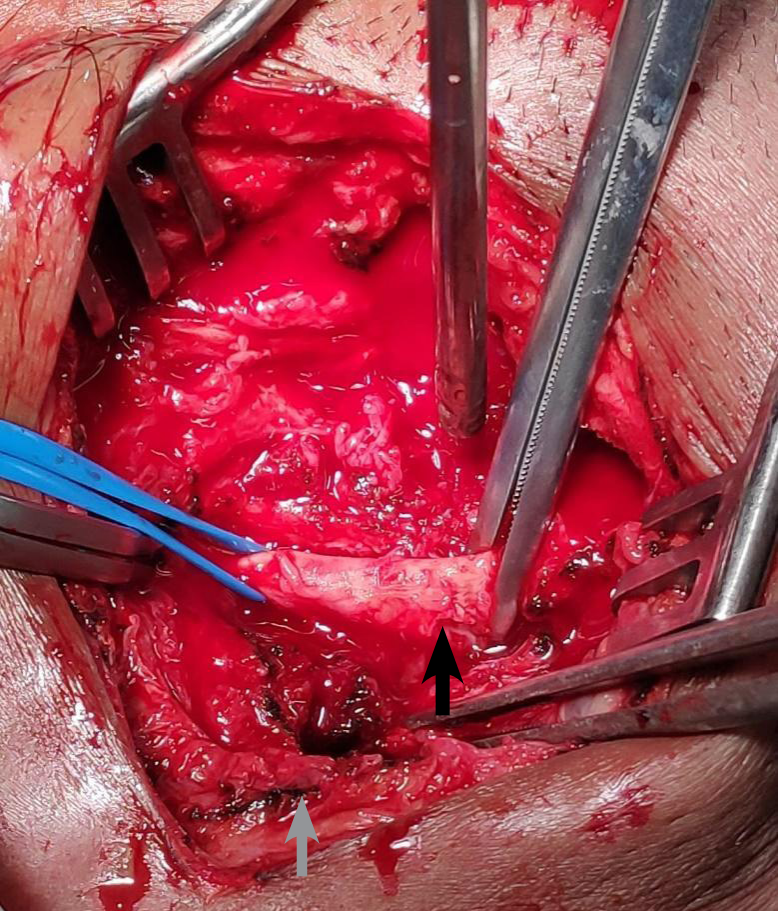
Intraoperative photograph showing the disruption in the right common femoral artery (black arrow) and site of pseudoaneurysm (grey arrow).

First, incisions for vascular control were given at two levels, one just proximal to the swelling and one just distal to the swelling. After securing the right common iliac artery and right common femoral artery, an incision was given over the swelling followed by exploration and hematoma evacuation. Then, the pseudoaneurysm was excised and a tear of 5 mm in the right common femoral artery was visualised (Figure 3). Repair of tear was performed with continuous suture using prolene 6-0 and a drain was placed in the subcutaneous plane at the surgical site before bandaging. Wound dressing was done on the second, fourth, and sixth postoperative days, and drain was removed on the fourth postoperative day. Postoperative hospital stay was uneventful and the patient was discharged on the seventh postoperative day with a healthy wound at the surgical site. The patient did not have any features of ischemia distal to the affected artery.

## DISCUSSION

Spontaneous femoral artery pseudoaneurysm is a rare occurrence with only few cases reported.^3-5^ Most cases of femoral artery pseudoaneurysms occur as a complication to endovascular procedures done through femoral access or after an anastomotic breakdown following arterial reconstructive surgery done at the femoral level and few cases occur following external trauma.^1,3^

Etiopathogenesis of spontaneous femoral artery pseudoaneurysm is not well understood. In older people, it has been proposed that atherosclerosis could lead to weakness of arterial walls predisposing to spontaneous rupture.^4^ In young individuals, reported causes of femoral artery pseudoaneurysm to include Ehler Danlos Syndrome (a connective tissue disorder) and Behcet's disease (an autoimmune, multisystemic vasculitis).^6,7^ Congenital abnormality of the arteries as a cause has also been proposed.^4^ In this case, the patient was young and without any comorbidity, there is no history of prior endovascular procedures, external trauma, or intravenous drug use. There were no clinical features of any other systemic illness. Duplex Ultrasound, CECT scan, and intraoperative examination did not reveal any pathology other than the disruption in the common femoral artery, the pseudoaneurysm, and the surrounding hematoma. So, this was labelled as a case of spontaneous common femoral artery pseudoaneurysm.

In most other cases of this disease, we find a pulsatile, tender swelling with bruits on auscultation.^1^ However, in this case, pulsations and bruits were not appreciated, probably because of the presence of a large surrounding hematoma measuring 9.2x8.8x5.2 cm as revealed by computed tomography imaging. Furthermore, no signs of pressure effect to surrounding structures were present. These findings combined with the spontaneous nature of onset and lack of further investigative tools may have led the community health worker in the village towards a wrongful diagnosis of cutaneous abscess followed by incision and drainage which could have led to devastating haemorrhage. Proper clinical examination including distal vascular examination and Doppler Ultrasonography, a simple outpatient investigation would have clearly prevented such procedure.

Treatment options include close observation for spontaneous thrombosis, ultrasound-guided compression, ultrasound-guided thrombin injection, surgical repair, and endovascular repair.^8,9^ In our case, persistent bleeding through the external communication of surrounding hematoma along with the potential of rupture due to the size of the lesion, open surgical repair was performed. Besides primary repair, other surgical options are peripheral bypass, reconstruction using grafts, and ligation of involved vessels as mentioned in the case series done at Kathmandu.^10^ In resource-limited settings, open surgical procedures are the mainstay of management of such cases.

Spontaneous formation of a femoral artery pseudoaneurysm as in this case is a rare occurrence. This case serves as a reminder that the possibility of a femoral artery pseudoaneurysm should be kept in mind while evaluating a swelling in the inguinal region. Also, alternative diagnoses should be ruled out before incising a suspected abscess.
